# Evaluation of cerebrovascular reactivity in chronic hepatitis C patients using transcranial color Doppler

**DOI:** 10.1371/journal.pone.0218206

**Published:** 2019-06-11

**Authors:** Mirela Pavicic Ivelja, Ivo Ivic, Kresimir Dolic, Antonio Mestrovic, Nikola Perkovic, Stipan Jankovic

**Affiliations:** 1 Department of Infectious Diseases, University Hospital Split, Split, Croatia; 2 Department of Radiology, University Hospital Split, Split, Croatia; 3 Department of Gastroenterology and Hepatology, University Hospital Split, Split, Croatia; Weill Cornell Medicine-Qatar, QATAR

## Abstract

Hepatitis C viral (HCV) infection is associated with systemic inflammation and metabolic complications that might predispose patients to atherosclerosis, including cerebrovascular atherosclerosis. The aim of this study was to assess cerebrovascular reactivity in patients with chronic hepatitis C. Seventeen patients with chronic hepatitis C infection, as well as 11 healthy blood donors in the control group, were assessed for cerebrovascular reactivity according to the well-established breath-holding test that uses the transcranial color Doppler for measurement of blood flow velocity. Results obtained during the breath-holding revealed significantly lower average peak systolic (AvPS start, *P* = 0.018), end-diastolic (AvED start, *P* = 0.031) and mean velocity values at the very beginning of the breath-holding procedure (AvmeanV start, *P* = 0.02), as well as a lower mean peak systolic velocity at the end of the breath-holding test (AvPS max, *P* = 0.02) in the hepatitis C group. Vascular reactivity values, calculated as the breath-holding index, were also significantly lower (*P* = 0.045) in the hepatitis C group. In conclusion, the results of this study suggest an association between chronic HCV infection and altered cerebrovascular reactivity which may ultimately have an unfavorable effect on cerebrovascular hemodynamics and lead to increased risk of cerebrovascular diseases.

## Introduction

Hepatitis C virus (HCV) infection is a major global health problem. There are 170 million people worldwide chronically infected with HCV with 3–4 million newly infected each year. Each year, 350,000 deaths occur due to all HCV-related complications. The major burden of HCV infection comes from sequelae of chronic infection, such as liver cirrhosis and hepatocellular cancer [1].

Cerebrovascular disease is the second leading cause of death worldwide; posing a significant health burden in most industrialized countries. Conventional risk factors for cerebrovascular disease include cigarette smoking, alcohol consumption, obesity, hyperlipidemia, hypertension and diabetes. In addition to the aforementioned, new risk factors such as infectious agents, have been documented. [2,3] HCV infection is associated with systemic inflammation and metabolic complications that might predispose patients to atherosclerosis, including cerebrovascular atherosclerosis. Chronic HCV infection has been associated with ultrasonographically defined carotid intima-media thickness or plaques, which are predictors for cerebrovascular disease. However, these studies could not verify a significant association between HCV infection and cerebrovascular disease, thus their link remained controversial. [4–7] Due to cerebral autoregulatory mechanisms, healthy brain arteries are capable of maintaining a constant cerebral blood supply despite changes in cerebral perfusion. Transcranial color Doppler (TCCD) enables a noninvasive evaluation of the main basal intracranial arteries, especially with established vascular malformations and vascular procedures monitoring. TCCD provides information on the systolic, diastolic and mean blood flow velocities (BFV) of the main basal intracranial arteries. Parameters related to cerebrovascular resistance include Gosling’s pulsatility index (PI), a measure of the BFV variability, and the resistance index (RI), or Pourcelot index, which reflects distal microvascular circulation resistance against the arterial flow. [8] Cerebral vasoreactivity assessment can be done by measuring BFV changes in response to vasodilatory stimulation such as during CO_2_ inhalation, breath-holding or acetazolamide administration. The breath holding test, in combination with TCCD, provides a well-tolerated, real-time screening method to study cerebrovascular hemodynamics and reactivity. [9] Finally, this method provides information regarding the reserve capacity of cerebral circulation, with respect to the adaption capability of vessels in response to systemic modification or brain metabolic activity demanding changes in cerebral blood flow. The reduced adaptability of vessels was found to be a predisposing condition for cerebrovascular disease. [10–14]

In previous studies, chronic HCV infection was found to be associated with a higher prevalence of stroke [15–17]. The aim of our study was to assess cerebrovascular reactivity, as an indicator of cerebrovascular disease, in patients with chronic HCV infection using TCCD and the breath-holding method.

## Materials and methods

We performed an cross-sectional observational study. The study was approved by the local institutional review board (University Hospital Split, Croatia) and written informed consent was obtained from all subjects. Participants were Caucasian adults divided into two groups: group one included 17 chronic hepatitis C patients, and group two consisted of 11 healthy blood donor volunteers. The patients´ history included: past and current diseases, as well as alcohol consumption and smoking. Exclusion criteria were: history of hypertension, diabetes mellitus, cirrhosis, cerebrovascular disease, hematologic disease, chronic heart disease or cancer, heavier alcohol consumption defined as drinking more than 7 drinks per week for women and more than 14 drinks per week for men [18–20], as well as using hormonal therapy, nitrates, β-blocking agents, calcium channel blockers, anticoagulants and vasodilatory drugs. Because of inadequate insonation of their middle cerebral artery (MCA), two of participants, one from each group, were excluded from the study. Concerning important sociodemographic variables, we found no statistically significant difference between the two groups (Table 1).

**Table 1:** Sociodemographic and clinical characteristics of of chronic hepatitis C and control group participants.

Participants’ characteristics	Hepatitis C group (N = 16)	Control group (N = 10)	Statistics
	No (%)	No (%)	
Male	12 (75)	10 (100)	Fisher’s exact *P* = 0.136
No alcohol consumption	16 (100)	10 (100)	NA†
Smokers	11 (69)	3 (30%)	Fisher’s exact *P* = 0.105
Right-handed	15 (94)	10 (100)	Fisher’s exact *P* = 1.000
Positive family history^‡^	1 (7)	0 (0)	Fisher’s exact *P* = 1.000
	Median (IQR)	Median (IQR)	
Age (year)	37,50 (33–42)	38,00 (27–41)	Mann-Whitney *P* = 0.618
Weight (kg)	85,00 (72–95)	90,00 (84–101)	Mann-Whitney *P* = 0.237
Height (m)	1,83 (1,75–1,92)	1,88 (1,85–1,94)	Mann-Whitney *P* = 0.238
BMI	24,98 (19,92–27,17)	24,84 (22,66–28,57)	Mann-Whitney *P* = 0.474
Estimated duration of hepatitis (year)	15,00 (9,5–20)	NA†	NA†

^†^ NA: not applicable

^‡^ Positive family history: cerebrovascular and/or cardiovascular disease.

We performed all measurements using the Acuson X500 Ultrasound system (Siemens, Erlangen, Germany) with a P4-2 (2–4 MHz frequency) transducer. Participants were examined during the afternoon in a quiet room while lying in a comfortable supine position without any visual and auditory stimulation after 5 minutes of bedrest. After measuring participants’ blood pressure, the arteries of the dominant side of the circle of Willis were insonated through the temporal bone window by standard protocol with special focus on the MCA. The MCA measurements in our study included peak systolic velocity (PS), end diastolic velocity (ED), mean velocity, RI and PI. Initial measurements were performed at rest. Then, following normal inspiration to exclude the Vasalva maneuver, we requested that participants hold their breath for as long as they could. The mean velocity (Vmean) of the MCA was continuously monitored during the breath-holding period and all measurements were done at the beginning of breath-holding and at the end of the procedure. We recorded the mean blood velocity at the end of breath-holding period as Vmax. The time of breath-holding (TBH) was also recorded to be used for further calculations. The entire procedure and all measurements were repeated after a 3 to 4 minute resting period and we determined the mean values of each variable in both measurements. Vascular reactivity was determined by calculating the breath-holding index (BHI) as a ratio between the percentage of increase of Vmean during breath holding and the duration (seconds) of breath-holding. [9]

### Statistical analysis

Statistical analysis was performed using the Statistical Package for the Social Sciences (version 16.0; SPSS, Chicago, Illinois). We used the Fisher exact and Mann-Whitney U tests for descriptive statistics. The Friedman test was used to assess differences in flow parameters measured in subjects at three different time points. The significance level was set at 0.05.

## Results

There were no significant differences in PS velocity, ED velocity, mean velocity, RI and PI between the two groups at rest. Additionally, there was no significant difference in average breath-holding time. Participants in the hepatitis C group compared to the controls had significantly lower average values of peak systolic (AvPS start, *P* = 0.018), end-diastolic (AvED start, *P* = 0.031) and mean velocity (Avmean Vstart, *P* = 0.02) at the very beginning of the breath-holding procedure, as well as of peak systolic velocity at the end of the breath-holding test (AvPS max *P* = 0.02). The BHI was significantly lower in the hepatitis C group (Table 2).

**Table 2:** Results of transcranial color Doppler measurements at rest and during the breath-holding test in chronic hepatitis C and control group.

Variable (unit)	Hepatitis C, N = 16		Control, N = 10		*P* value*
	Median	(IQR)	Median	(IQR)	
Syst (mmHg)	127,5	(120–130)	127,5	(120,00–131,25)	0.913
Diast (mmHg)	77,5	(70–80)	75	(65,00–82,50)	0.807
MCA RI rest (cm/s)	0,52	(0,49–0,55)	0,535	(0,49–0,60)	0.302
MCA PI rest (cm/s)	0,76	(0,69–0,87)	0,85	(0,69–0,98)	0.187
MCA PS rest (cm/s)	105,9	(88,40–131,23)	128,45	(111,98–148,78)	0.073
MCA ED rest (cm/s)	49,8	(44,85–65,38)	57,05	(49,35–67,05)	0.246
MCA S/D rest (cm/s)	2,085	(1,97–2,23)	2,155	(1,97–2,50)	0.329
MCA mean V rest (cm/s)	71,15	(59,63–92,63)	81,65	(72,60–95,45)	0.188
AvTBH (s)	37,00	(28,50–45,13)	27,50	(25,88–38,50)	0.292
AvRIstart	0,51	(0,48–0,55)	0,49	(0,46–0,56)	0.509
AvPIstart	0,74	(0,68–0,84)	0,76	(0,67–0,84)	0.932
AvPSstart (cm/s)	99,83	(79,26–115,85)	132,48	(118,50–140,95)	0.018^†^
AvEDstart (cm/s)	48,50	(39,64–59,48)	61,98	(53,35–70,30)	0.031^†^
AvS/Dstart (cm/s)	2,04	(1,94–2,22)	1,97	(1,87–2,25)	0.477
AvMeanVstart (cm/s)	71,10	(55,03–76,05)	85,73	(80,58–95,74)	0.02^†^
AvRImax	0,45	(0,39–0,48)	0,46	(0,42–0,54)	0.492
AvPImax	0,60	(0,50–0,67)	0,63	(0,54–0,79)	0.46
AvPSmax (cm/s)	119,85	(97,13–158,08)	163,30	(137,29–171,60)	0.02^†^
AvEDmax (cm/s)	74,73	(50,58–86,49)	79,05	(73,84–94,50)	0.155
AvS/Dmax (cm/s)	1,81	(1,64–1,94)	1,85	(1,73–2,15)	0.527
AvMeanVmax (cm/s)	93,78	(69,34–119,70)	110,25	(100,13–123,64)	0.065
BHI	0,51	(0,48–0,55)	0,968979	(0,67–1,37)	0.045^†^

* Mann-Whitney U test;

^†^ Significance level at 0.05

Syst—systolic blood pressure at rest, Dyast—diastolic blood pressure at rest, MCA RI rest—Resistance Index at rest, MCA PI rest—Pulsatility Index at rest, MCA PS rest—peek systolic velocity at rest, MCA ED rest—end-diastolic velocity at rest, MCA S/D rest—systolic-diastolic ratio at rest, MCA mean V rest—mean velocity at rest, AvTBH—average time of breath-holding, AvRIstart—average Resistance Index at the start of breath holding, AvPIstart- average Pulsatility index at the start of breath-holding, AvPSstart—average Peek systolic velocity at the start of breath-holding, AvEDstart—average end-diastolic velocity at the start of breath-holding, AvS/Dstart- average systolic-diastolic ratio at the start of breath-holding, AvMeanVstart—average mean velocity at the start of breath-holding, AvRImax—average Resistance Index at the end of breath-holding, AvPImax—average Pulsatility index at the end of breath-holding, AvPSmax-average peek systolic velocity at the end of breath-holding, AvEDmax—average end-diastolic velocity at the end of breath-holding, AvS/Dmax—average systolic-diastolic ratio at the end of breath-holding, AvMeanVmax—average mean velocity at the end of breath-holding, BHI—breath-holding index.

### Peak systolic velocity at the start of the breath-holding test (PS start)

**Fig 1:**
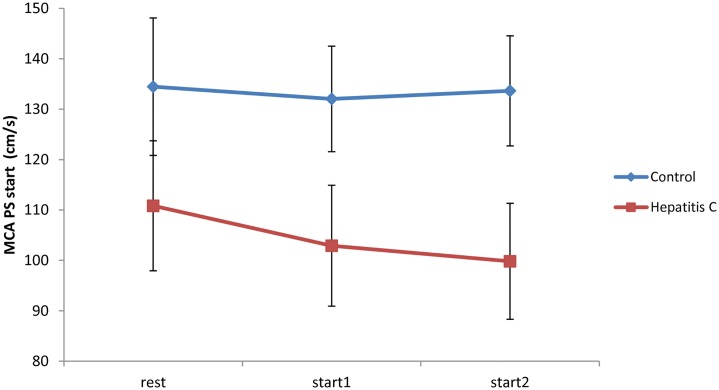
Changes in peak systolic velocity (PS) velocity at rest and at the start (start1 and start2) of two consecutive breath-holding tests.

PS velocity determined at rest in the control group did not significantly differ from values obtained at the start (start1 and start2) of two consecutive breath-holding tests (Friedman test, *P* = 0.459). In contrast, the hepatitis C group showed a significant decline in PS start velocity during the test (Friedman test, *P* = 0.002). Post-hoc analysis showed that this significance originated from the significant decline of PS start2 velocity (post hoc test, *P* = 0.001) (Fig 1).

### Peak systolic velocity at the end of the breath-holding test (PSmax)

**Fig 2:**
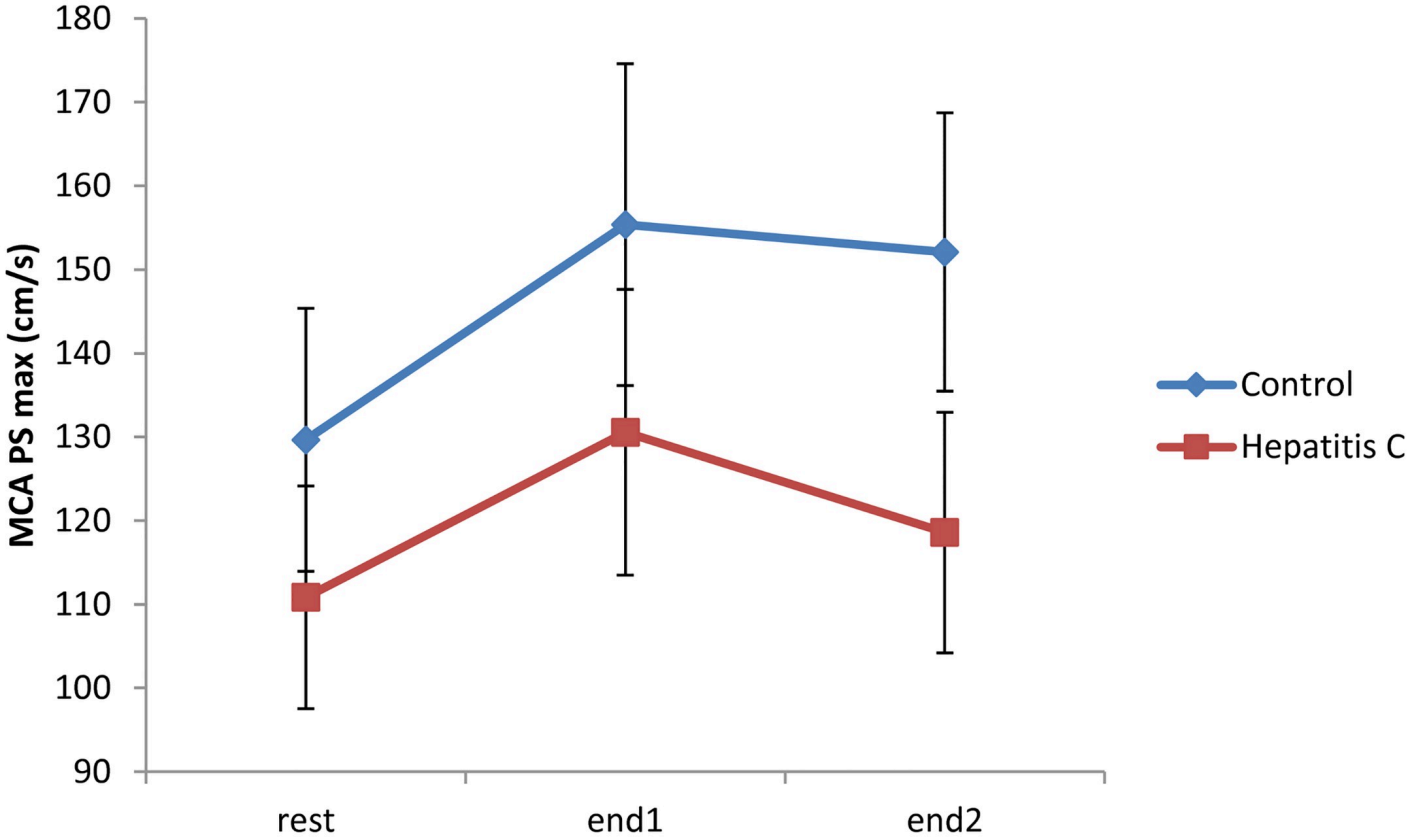
Changes in peak systolic velocity at the end of the breath-holding test (PSmax) velocity at rest and at the end of two consecutive breath-holding tests (end1 and end2).

The Friedman test was used to determine differences in PSmax values between two consecutive breath-holding tests and the rest period. PSmax during both consecutive breath-holding tests significantly increased from the rest period for both the healthy control and hepatitis C groups, (*P*< 0.002 for both comparisons), with a *P*<0.016 for the control group (post hoc tests). In contrast, the PSmax in the hepatitis C group for the first breath-holding test was significantly higher than in the remaining tests (post hoc, *P* = 0.001), while the PSmax of the second breath-holding test was comparable to the PSmax in the remaining tests (post hoc, *P* = 0,335) (Fig 2).

PSmax in the hepatitis C group for the first breath-holding test was significantly higher than in the rest period (post hoc, *P* = 0.001), whereas PSmax values in the second breath-holding test were similar to those in the rest period (post hoc, *P* = 0.335).

### Breath-holding index (BHI)

**Fig 3:**
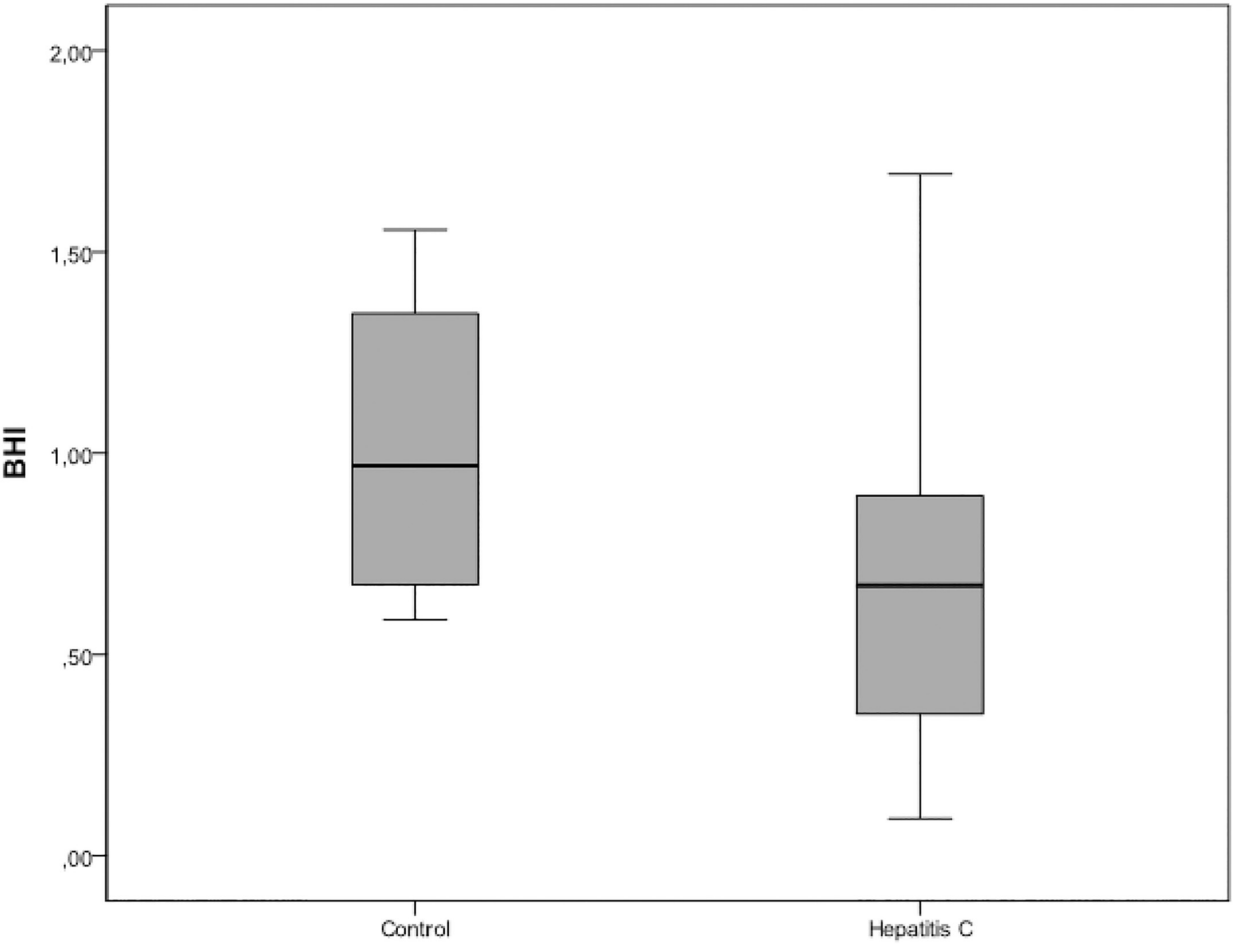
Distribution of breath-holding index (BHI) values in the hepatitis C and control groups.

Calculated BHI values were significantly lower in the hepatitis C group than in the healthy control group (*P* = 0.045) (Fig 3).

## Discussion

Studies using provocation methods, such as the breath-holding test, to estimate the reactivity of cerebral vessels in adult patients with chronic HCV infection have not yet been published. In our study, we found no significant differences between chronic hepatitis C patients and healthy volunteers at the resting state (Table 2). However, in comparison to the control group, we found that patients with chronic HCV infection had significantly lower average values of peak systolic, end-diastolic and mean velocity at the beginning of the breath-holding procedure. Also, they had a lower average peak systolic velocity at the end of the breath-holding test and lower BHI values than the control group (Table 2, Fig 3). Previous studies have shown that the reduction of vascular adaptability is manifested by a likewise reduction in vasodilatatory capacity of the cerebral arteries, a precursor condition for the development of cerebrovascular disease [21–25]. Furthermore, compared with resting state values, we have also found a significant deceleration of the average PS start velocity, in particular during the second breath-holding test as the main source of this difference (Fig 1). Similar results were obtained by measuring the average PSmax velocity at the end of the breath-holding period. Both groups showed significant changes in PSmax (*P*<0,002, for both comparisons). However, in the control group PSmax values during breath-holding tests were significantly higher than in the rest period (*P*<0.016, post hoc tests). Notably, PSmax in the first breath-holding procedure was significantly higher than in the resting state in the HCV group, while PS max in the second breath-holding procedure was comparable to the resting state PSmax (Fig 2). Additionally, our results support the possibility of an altered adaptive capacity of intracranial blood vessels in patients with HCV infection. Differences between the two groups in the current study arise mainly from HCV patients’ weaker response to hypercapnia in the second breath-holding maneuver, probably because of exhaustion of intracranial vessels and their incapacity to respond to the more demanding metabolic requirements of the brain.

Cerebrovascular disease is etiologically heterogeneous and is associated with a variety of underlying diseases and risk factors: hypertension, diabetes mellitus, cardiac diseases, smoking, alcohol consumption, unhealthy diet, abdominal obesity, lack of exercise, psychosocial stress, and depression. However, young people who have had a stroke often do not experience any of these factors. There is increasing evidence indicating that various acute and chronic infections such as *Chlamydia pneumoniae*, human cytomegalovirus, *Helicobacter pylori*, influenza virus, periodontal pathogens, etc., contribute to the development of atherosclerosis through different mechanisms [26].

Although the majority of researches have pointed out chronic HCV infection as a risk factor for cerebrovascular disease [15–17], not all studies support this suggestion. Younossi et al. [27] did not show an association between chronic HCV infection and cerebrovascular disease. Adinolfi et al. [16] found a higher prevalence of HCV in patients with stroke than in the control group (26.8% vs 6.6%, P = 0.0001). Moreover, HCV-positive patients with stroke were younger and with fewer cerebrovascular disease risk factors than HCV-negative patients. A meta-analysis performed by Huang et al. also indicated that HCV infection is associated with carotid atherosclerosis; independent of classical risk factors. [28] Although the above mentioned data suggests that HCV infection may increase the risk of atherosclerosis and cerebrovascular disease, the number of studies is still insufficient, requiring cautious interpretation of their results. Sorrentino et al. in 2004 [29], as well as Targher et al. in 2006 [30], have found circulation changes in hepatitis C patients’ ocular and carotid arteries, respectively, but most of their participants had liver cirrhosis. As advanced stage liver fibrosis, regardless of its etiology, is characterized by hemodynamic alterations, cirrhotic patients were not included in our study.

The precise pathogenetic mechanism that could explain the role of chronic HCV infection in atherosclerosis has not been thoroughly investigated. In general, it is assumed that chronic infection induces prolonged systemic inflammatory response which damages the blood vessel endothelium. A damaged endothelium favors the development of atherosclerostic plaques that progress and destabilize [31,32]. A chronic HCV infection promotes both hepatic and systemic inflammation, which could be triggered by the HCV either directly or indirectly. Their study concerning HCV RNA sequences in the carotid plaques led Boddi and colleagues to hypothesize that HCV may have a direct pro-atherogenic role by stimulating arterial inflammation [33]. Finally, Hsu et al. in their large retrospective study [34] concluded that interferon-based therapy may reduce the long-term risk of stroke in patients with chronic HCV infection. Therefore, it would be of interest to examine the impact of antiviral therapy on cerebrovascular reactivity.

In conclusion, the results of our study suggest that patients with chronic hepatitis C have altered cerebrovascular reactivity. These negative effects on cerebrovascular hemodynamics could contribute, at least in part, to the increased risk of cerebrovascular disease. The breath-holding test and TCCD provide a simple non-invasive evaluation of cerebrovascular disease risk in patients with chronic hepatitis C and further studies with a larger number of participants should be performed. Future studies should also include post-treatment HCV patients in order to assess the impact of antiviral therapy on cerebrovascular reactivity.
